# Genitourinary Syndrome of Menopause: Number 3 - March 2022

**DOI:** 10.1055/s-0042-1748463

**Published:** 2022-05-16

**Authors:** Ana Lúcia Ribeiro Valadares, Jaime Kulak Junior, Lúcia Helena Simões da Costa Paiva, Elizabeth Jeha Nasser, Célia Regina da Silva, Eliana Aguiar Petri Nahas, Luiz Francisco Cintra Baccaro, Márcio Alexandre Hipólito Rodrigues, Marco Aurélio Albernaz, Maria Celeste Osório Wender, Maria Célia Mendes, Rita de Cassia de Maio Dardes, Rodolfo Strufaldi, Rogerio Cesar Bocardo, Luciano de Melo Pompei

**Affiliations:** 1 Universidade Estadual de Campinas, Campinas, SP, Brazil; 2 Universidade Federal do Paraná, Curitiba, PR, Brazil; 3 Universidade Estadual de Campinas, Campinas, SP, Brazil; 4 Departamento de Ginecologia e Obstetrícia, Faculdade de Medicina do ABC, Santo André, SP, Brazil; 5 Universidade Federal do Rio de Janeiro, Rio de Janeiro, RJ, Brazil; 6 Universidade Estadual Paulista “Júlio de Mesquita Filho”, Botucatu, SP, Brazil; 7 Universidade Estadual de Campinas, Campinas, SP, Brazil; 8 Universidade Federal de Minas Gerais, Belo Horizonte, MG, Brazil; 9 Universidade Federal de Goiás, Goiânia, GO, Brazil; 10 Universidade Federal do Rio Grande do Sul, Porto Alegre, RS, Brazil; 11 Faculdade de Medicina de Ribeirão Preto, Universidade de São Paulo, Ribeirão Preto, São Paulo, Brazil; 12 Departamento de Ginecologia, Escola Paulista de Medicina, Universidade Federal de São Paulo, São Paulo, SP, Brazil; 13 Departamento de Ginecologia e Obstetrícia, Faculdade de Medicina do ABC, Santo André, SP, Brazil; 14 Faculdade de Medicina de Jundiaí, Jundiaí, SP, Brazil; 15 Departamento de Ginecologia e Obstetrícia, Faculdade de Medicina do ABC, Santo André, SP, Brazil

## Key points

- Genitourinary syndrome is a complex chronic syndrome with multiple changes in genitourinary tissues in response to menopausal loss of estrogen.
- Symptoms range from mild to severe and may have effects on genitourinary and sexual function, relationships and quality of life.
- Treatments can be non-hormonal with lubricants and moisturizers or with vaginal or systemic estrogen therapy.
- A multidisciplinary approach may be needed when there are complex problems, including sexual dysfunction.
- The gynecologist is in a unique position to sensitively discuss the symptoms of genitourinary syndrome, advise, educate and treat appropriately, providing long-term follow-up.

## Recommendations

- The choice of therapy depends on the severity of symptoms, the efficacy and safety of treatments, and individual patient preference.
- Non-hormonal therapies with lubricants and moisturizers are effective for most women with mild symptoms of genitourinary syndrome.
- Vaginal estrogen therapy is the most effective standard treatment for menopausal genitourinary syndrome symptoms.
- In cases of moderate and severe symptoms, vaginal estrogen therapy or systemic hormonal therapy is recommended, with vaginal estrogen therapy being more effective.
- In open prospective studies or randomized trials with small samples, both the microablative fractional CO2 laser and the non-ablative Erbium 2940 nm laser have proven effective in the treatment of genitourinary syndrome.
- In women after breast cancer with genitourinary syndrome, first-line treatment is the use of lubricants and moisturizers. Prescription of vaginal estrogen therapy should be avoided; if indicated in particular cases, the severity of symptoms and the oncologist's agreement should be considered.
- In cases refractory to treatment, other etiologies should be evaluated, such as vulvodynia, pelvic floor dysfunction, or painful bladder syndrome.

## Background

Genitourinary syndrome of menopause (GSM), formerly called vulvovaginal atrophy (VVA), is a syndrome defined as a set of signs and symptoms resulting from estrogen deficiency in the female genitourinary tract, including the vagina, lips, urethra, and bladder. This syndrome includes genital symptoms of dryness, burning and irritation, urinary symptoms and conditions of dysuria, nocturia, urgency and recurrent urinary tract infections (UTIs) and sexual symptoms of dyspareunia and lack of lubrication. 1 Although it is more frequent in climacteric women, it can also occur in other situations of hypoestrogenism. 1

The genitourinary syndrome of menopause affects peri- and postmenopausal women, with a prevalence ranging from 36% to almost 90%. This condition is also present in pre-menopausal years, with a prevalence of 19% in women aged 40-45 years. Unlike other symptoms of menopause that are temporary and tend to decrease over time, if not treated properly, GSM tends to get worse with age and with longer duration of hypoestrogenism. 2

In postmenopausal women, 64% reported loss of libido and 58% avoided any sexual intimacy. In Brazil, 44% of women believe that vaginal symptoms compromise their self-esteem. Even those who are not sexually active can experience discomfort resulting from the symptoms. 3

Despite being very common symptoms, more than 70% of symptomatic women do not complain or report these symptoms to their physician. Studies also show that healthcare professionals do not always actively inquire about GSM and often do not prescribe any treatment for women. 4

These symptoms are directly related to the reduction in circulating estrogen levels after menopause. Estrogen receptors (ERs; both α and β) are present in the vagina, vulva, pelvic floor musculature, endopelvic fascia, urethra, and bladder trigone. As a result of the estrogen deficiency that occurs in the climacteric stage, histological and anatomical changes occur in urogenital tissues. These changes lead to reduced vaginal elasticity, increased vaginal pH, changes in the vaginal flora, decreased lubrication, vulnerability to irritation and physical trauma. With advancing age, these women become increasingly susceptible to suffering from genital and urinary symptoms. 1 4 5

## How is the diagnosis made?

Diagnosis is basically clinical and performed through anamnesis and gynecological examination ( Chart 1 ). The most commonly reported symptoms of genital atrophy are vaginal dryness (55%), dyspareunia (44%) and genital irritation (37%), with an impact on sexual function (59%). 6 Physicians should actively inquire about these complaints during the anamnesis because, as discussed above, many women may not spontaneously report these symptoms. Vaginal pH measurement with colorimetric tape, Vaginal Health Index and vaginal maturation index can also be performed.

**Chart 1.** Genital signs and symptoms on physical examination

**SYMPTOMS**	**SIGNS**
Vaginal dryness Irritation and genital burning Dyspareunia Decreased lubrication in sexual activity Vulvovaginal itching Discomfort and acute genital pain Abnormal vaginal discharge Postcoital bleeding	Pubic hair loss Fusion of the labia minora or synechiae Clitoris foreskin synechia Vaginal introitus stenosis Vaginal walls with pale mucosa, loss of roughness and elasticity, often friable and with petechiae that bleed easily on specular examination or Pap smear Shortened cervix Difficult visualization of the cervical orifice Shortened vagina and sometimes with stenosis Pale, dry, thinned vaginal epithelium Vaginal discharge: watery or purulent fluid Irregular erythema Vaginal petechiae vaginal pH ≥ 5
**URINARY**	
Urinary urgency Increased urinary frequency Nocturia Dysuria Recurrent urinary tract infections	Eversion or urethral prolapse Urethral meatus prominence

Source: Portman et al. 1

## What are the initial therapeutic options?

Lubricants and moisturizers are the initial first-line therapies recommended to relieve mild to moderate symptoms of GSM. 4

Vaginal lubricants are used on the partner's penis, vaginal opening, and the woman's vulva before and during intercourse, and can improve pain and ease vaginal penetration by decreasing friction during intercourse. Although these can improve vaginal discomfort and sexual pleasure, they do not have the ability to reverse atrophic changes in the vaginal mucosa. 7

Ideally, lubricants should have an osmolality of up to 380 mOsm/kg, but in clinical practice most lubricants exceed this value, with up to 1,200 mOsm/kg being acceptable. Hyperosmotic products can cause irritation and toxicity in the epithelial cells of the vaginal mucosa, which is not the case with water-based or silicone-based iso-osmotic products. They may also contain glycerin, glycerol, preservatives such as propylene glycol or parabens and microbicides. Microbicides can affect the lactobacilli population, decreasing the defense and protection against infections, although not all studies confirm this change in the vaginal flora. 7 8 Since there is not much research on the safety and adverse effects of lubricants, it is advisable to give preference to iso-osmotic lubricants and those physiologically similar to natural vaginal secretions. 4 8

Vaginal moisturizers are long-acting, non-hormonal products that, unlike lubricants, should be used regularly two to three times a week. Such products contain a polymer that adheres to the vaginal wall for three days and binds to water molecules, which are then released into the vaginal tissue. They are bioadhesives that contain acids such as polyacrylic or hyaluronic or polycarbophil. In some moisturizers, lactic acid is also added to make the pH more acidic. 4 9 In a recent systematic review, only five clinical trials with hyaluronic acid were identified and included, totaling 335 women. It showed that the efficacy and tolerability profile of hyaluronic acid are similar to those of vaginal estrogen, thereby making it a good non-hormonal alternative for the treatment of vaginal atrophy of postmenopausal women. 9

As data about the effectiveness of lubricants and moisturizers from clinical trials are scarce, most guidelines consider topical estrogen to be superior to vaginal moisturizers, being the standard treatment. 4 10 11

## How is it handled in persistent symptoms?

The administration of vaginal estrogen therapy (ET) favors the reestablishment of vulvovaginal trophism and is currently the best treatment for this condition. Among the options for vaginal use available in Brazil, we have 17-β-estradiol, promestriene and estriol. 4 The recommendation for use of any of these formulations is an initial intravaginal application for 14 days in the evening, followed by a maintenance application two to three times a week while symptoms persist. 4 Energy, laser, and radio frequency-based therapies are other options. 4

## Vaginal estrogen therapy

### 17-β-estradiol

Vaginal tablets with 10 mcg of estradiol show efficient and safe results. In a randomized double-blind study, 309 menopausal women were treated with vaginal tablets containing 10 mcg of estradiol. After 12 weeks, there was a significant improvement in the percentage of parabasal and superficial cells and a reduction in vaginal pH, compared to the placebo group. Clinical improvement was evidenced after four weeks, persisting until the end of the study, at 52 weeks. 12 Another study evaluated endometrial safety in 336 women over 52 weeks, without finding an increase in endometrial thickness. 13 Additional safety studies evaluating the endometrium through biopsies have been published, without finding an increase in endometrial hyperplasia or carcinoma. 14

The use of estradiol vaginal tablets at a dose of 10 mcg offers local action without significant systemic absorption, and its concentration remains low and stable over time. One study showed that estradiol concentrations remained between 2.44-12.08 pg/mL after 52 weeks of evaluation, being comparable to those in postmenopausal women without treatment. 15

After evaluating the risks and benefits, the use of estradiol vaginal tablets at a dose of 10 mcg proved to be effective and safe, becoming the first-line treatment of GSM, especially in patients without other climacteric symptoms. 4

### Promestriene

It is a synthetic estrogen obtained by double esterification of estradiol used vaginally with local effect, which has not had systemic estrogenic effects. Therefore, it may be a first-line option for women who need minimal vaginal absorption. 16

A Brazilian systematic review including nine short-term studies (14-40 days in eight of them) and with a relatively small number of patients demonstrated improvement in symptoms (total or reduced intensity). 16 Another uncontrolled clinical trial study demonstrated improvement in vaginal atrophy (p<0.01) and an increase in vaginal health score after treatment with promestriene (p<0.01). 17

Evaluation of prescriptions for promestriene for nearly 40 years in 34 countries has shown very low vaginal absorption even after 4-6 months of therapeutic doses. 18

### Estriol

It is an estrogen derived from the metabolism of estrone and estradiol in the liver, and its affinity with estrogen replacement RE is around 10% to 15% of the estradiol affinity. Even with this difference in activity, estriol has been used safely and effectively for several years in the treatment of GSM. In six studies included in a systematic review comparing vaginal estradiol and high-dose estriol (0.5-1.0 mg/day), these products were found to be equally effective in relieving the subjective and objective symptoms of GSM. 19

In women treated with low-dose vaginal estrogen preparations, the addition of progestin is generally not necessary for endometrial protection, although patients who experience postmenopausal bleeding while undergoing treatment should be evaluated as appropriate.

### Energy, laser and radio frequency-based therapies

Laser can be considered a therapeutic option that enables women to avoid hormonal interventions in the treatment of GSM. Microablative fractional CO2 laser or non-ablative YAG Erbium vaginal laser (VEL) can be used. 20

Treatment with CO2 laser or VEL is an ambulatory procedure generally consisting of a series of three to four applications at four to six-weeks intervals. 20 21

Radiofrequency with a non-ablative effect is another treatment with electromagnetic energy that has also been studied for GSM. 20

A recent systematic review included 49 studies that evaluated physical methods of treating GSM. Of these, 37 were about the CO2 laser, of which only four were randomized clinical trials. Ten studies on the Erbium laser were included and only one was a randomized clinical trial. Of the two radiofrequency studies, one was a double-blind, randomized, controlled clinical trial, and the other was a prospective open-label trial. This review suggested that laser therapy is effective and safe in postmenopausal women with GSM. There is still little evidence to support the hypothesis that radiofrequency therapy is safe and effective for GSM 22

Regarding vaginal laser therapy in breast cancer survivors, studies suggest improved vaginal health in this group. 23

However, it is important to remember that most studies with energy-based equipment were not simulation or placebo controlled and included a small number of women with a short follow-up time. 22 23

### Other drug therapies not available in Brazil

Other therapies include vaginal testosterone, vaginal dehydroepiandrosterone (DHEA), and oral ospemifene. Although these treatments are not marketed in Brazil, they have proven effective in treating the vaginal symptoms of GSM. 4

## Is there evidence of vaginal ET in the relief of urinary symptoms?

A systematic review showed that vaginal ET, compared with placebo, significantly reduced urinary urgency, frequency or nocturia, and stress and urge urinary incontinence. For recurrent UTIs, some studies show a reduction in the frequency of infections, although with less evidence. 24 25

## What to do in cases refractory to treatment?

### Evaluate other etiologies

In cases of treatment failure with vaginal ET, after confirming the correct use of medication, evaluate other etiologies such as vulvodynia, pelvic floor dysfunction 26 or painful bladder syndrome. 4 26

### Associate pelvic physiotherapy

Patients with GSM who do not respond to vaginal treatments or have contraindications to hormone therapy should be referred to a pelvic physiotherapist for evaluation and treatment. 26

### Use vaginal dilators

Dilators may be useful for patients with GSM who present contraindications to estrogen therapy and have not been able to improve with moisturizers and lubricants. They are also useful for patients with introital stenosis or post-radiotherapy vaginal shortening and/or stenosis. 4 26

## What are the special situations for using ET?

### Asymptomatic patients

The treatment of a patient with asymptomatic vaginal atrophy is indicated in some clinical contexts:

- In the preoperative period of vulvovaginal surgery. Vaginal ET is used for 14 days before the date of surgery in order to optimize the identification of tissue planes and promote scaring of the wound; 27
- In the presence of pelvic organ prolapse. In patients with pelvic organ prolapse and pessary users, vaginal ET prevents vaginal abrasions caused by its use; 26
- A third situation is that of patients with significant vaginal atrophy on physical examination, who have never had vaginal intercourse or have been without sexual activity for some time, and are planning to initiate/resume intercourse with vaginal penetration. Treatment must be offered before the patient has vaginal intercourse in order to avoid painful intercourse and facilitate the resumption of sexual life. 26

### Patient with severe anatomical changes

Hypoestrogenism can cause moderate to severe anatomical distortion in the vulva and vagina. This can include adhesion of the labia majora, introital narrowing, vaginal shortening or stenosis. Treatment should only be carried out if the patient wishes or if she is symptomatic. 4 26 Differential diagnosis must be made with vulvovaginal dermatosis or malignant alterations. 1 4

For patients with labial adhesion who are symptomatic or wish to resume sexual activity with vaginal penetration, first-line therapy is the use of estrogen in the area of agglutination and application of slight pressure with finger. 26

In cases of symptomatic introital narrowing and/or vaginal shortening or stenosis, first-line therapy is vaginal ET associated with the use of graduated vaginal dilators. Estrogen cream can be applied to the dilator. In very serious cases and as exception therapy, if there is no improvement with vaginal ET, surgical options should be discussed with the patient and performed by a trained professional. 26

### Breast cancer patients

For women with a personal history of breast cancer, first-line treatment is performed with vaginal lubricants and moisturizers. Prescription of vaginal ET should be avoided and performed only in specific situations, depending on the type and characteristics of the cancer, with prior information and consent, in a decision taken in conjunction with the woman's oncologist, evaluating the risks and benefits. 28 29 30

## Final considerations

The diagnosis of GSM is of paramount importance, as it can negatively affect women's lives. Health professionals need to be more proactive and ask questions during the anamnesis about the presence of symptoms and prescribe treatment when indicated. There is a range of therapies available. For milder symptoms, lubricants and moisturizers are first-line treatments and for moderate to severe symptoms, vaginal estrogen therapy is the most effective and evident. Treatment with low-dose vaginal ET should be continued for as long as symptoms persist, with adequate patient follow-up. New technologies with the use of energy-based treatments such as vaginal laser and radiofrequency have proven effective and safe when used by properly trained professionals, although many aspects still need to be clarified.

National Specialized Commission on Climateric of the Brazilian Federation of Gynecology and Obstetrics Associations (FEBRASGO)

President:

Luciano de Melo Pompei

Vice-President:

Lúcia Helena Simões da Costa Paiva

Secretary:

Elizabeth Jeha Nasser

Members:

Ana Lúcia Ribeiro Valadares

Célia Regina da Silva

Eliana Aguiar Petri Nahas

Jaime Kulak Junior

Luiz Francisco Cintra Baccaro

Márcio Alexandre Hipólito Rodrigues

Marco Aurélio Albernaz

Maria Celeste Osório Wender

Maria Célia Mendes

Rita de Cassia de Maio Dardis

Rodolfo Strufaldi

Rogerio Cesar Bocardo

## References

[1] Vulvovaginal Atrophy Terminology Consensus Conference Panel Portman D J Gass M L Genitourinary syndrome of menopause: new terminology for vulvovaginal atrophy from the International Society for the Study of Women's Sexual Health and the North American Menopause Society Menopause 2014 21 10 1063 8 10.1097/GME.0000000000000329 25160739

[2] Cagnacci A Xholli A Sclauzero M Venier M Palma F Gambacciani M Vaginal atrophy across the menopausal age: results from the ANGEL study Climacteric 2019 22 01 85 9 10.1080/13697137.2018.1529748 30601037

[3] Nappi R E de Melo N R Martino M Celis-González C Villaseca P Röhrich S Vaginal Health: Insights, Views & Attitudes (VIVA-LATAM): results from a survey in Latin America Climacteric 2018 21 04 397 403 10.1080/13697137.2018.1461826 29741110

[4] The 2020 genitourinary syndrome of menopause position statement of The North American Menopause Society Menopause 2020 27 09 976 92 10.1097/GME.0000000000001609 32852449

[5] Frank S M Ziegler C Kokot-Kierepa M Maamari R Nappi R E Vaginal Health: Insights, Views & Attitudes (VIVA) survey – Canadian cohort Menopause Int 2013 19 01 20 7 10.1258/mi.2012.012034 23201626

[6] Kingsberg S A Wysocki S Magnus L Krychman M L Vulvar and vaginal atrophy in postmenopausal women: findings from the REVIVE (REal Women's VIews of Treatment Options for Menopausal Vaginal ChangEs) survey J Sex Med 2013 10 07 1790 9 10.1111/jsm.12190 23679050

[7] Edwards D Panay N Treating vulvovaginal atrophy/genitourinary syndrome of menopause: how important is vaginal lubricant and moisturizer composition? Climacteric 2016 19 02 151 61 10.3109/13697137.2015.1124259 26707589 PMC4819835

[8] Potter N Panay N Vaginal lubricants and moisturizers: a review into use, efficacy, and safety Climacteric 2021 24 01 19 24 10.1080/13697137.2020.1820478 32990054

[9] Dos Santos C C Uggioni M L Colonetti T Colonetti L Grande A J Da Rosa M I Hyaluronic acid in postmenopause vaginal atrophy: a systematic review J Sex Med 2021 18 01 156 66 10.1016/j.jsxm.2020.10.016 33293236

[10] IMS Writing Group Baber R J Panay N Fenton A 2016 IMS Recommendations on women's midlife health and menopause hormone therapy Climacteric 2016 19 02 109 50 10.3109/13697137.2015.1129166 26872610

[11] Donders G G Ruban K Bellen G Grinceviciene S Pharmacotherapy for the treatment of vaginal atrophy Expert Opin Pharmacother 2019 20 07 821 35 10.1080/14656566.2019.1574752 30897020

[12] Nachtigall L Lang E Gut R Utian W Archer D F Simon J An ultra-low dose (10μg) estradiol vaginal tablet improves signs and symptoms associated with vaginal atrophy Fertil Steril 2008 90Suppl:S250 10.1016/j.fertnstert.2008.07.1315 18978105

[13] VAG-1748 trial investigators Ulrich L S Naessen T Elia D Goldstein J A Eugster-Hausmann M Endometrial safety of ultra-low-dose Vagifem 10 microg in postmenopausal women with vaginal atrophy Climacteric 2010 13 03 228 37 10.3109/13697137.2010.481058 20423243

[14] Simon J Nachtigall L Ulrich L G Eugster-Hausmann M Gut R Endometrial safety of ultra-low-dose estradiol vaginal tablets Obstet Gynecol 2010 116 04 876 83 10.1097/AOG.0b013e3181f386bb 20859151

[15] Eugster-Hausmann M Waitzinger J Lehnick D Minimized estradiol absorption with ultra-low-dose 10 microg 17beta-estradiol vaginal tablets Climacteric 2010 13 03 219 27 10.3109/13697137.2010.483297 20423242

[16] Pompei L M Fernandes C E Melo N R Promestrieno no tratamento da atrofia vulvovaginal: revisão sistemática Femina 2010 38 07 359 65

[17] Sun A J Lin S Q Jing L H Wang Z Y Ye J L Zhang Y [Safety of promestriene capsule used in postmenopausal atrophic vaginitis] Zhonghua Fu Chan Ke Za Zhi 2009 44 08 593 6. Chinese 20003787

[18] Del Pup L Di Francia R Cavaliere C Facchini G Giorda G De Paoli P Promestriene, a specific topic estrogen. Review of 40 years of vaginal atrophy treatment: is it safe even in cancer patients? Anticancer Drugs 2013 24 10 989 98 10.1097/CAD.0b013e328365288e 24080714

[19] Biehl C Plotsker O Mirkin S A systematic review of the efficacy and safety of vaginal estrogen products for the treatment of genitourinary syndrome of menopause Menopause 2019 26 04 431 53 10.1097/GME.0000000000001221 30363010

[20] Wańczyk-Baszak J Woźniak S Milejski B Paszkowski T Genitourinary syndrome of menopause treatment using lasers and temperature-controlled radiofrequency Prz Menopauzalny 2018 17 04 180 4 10.5114/pm.2018.81743 30766466 PMC6372847

[21] Tadir Y Gaspar A Lev-Sagie A Alexiades M Alinsod R Bader A Light and energy based therapeutics for genitourinary syndrome of menopause: consensus and controversies Lasers Surg Med 2017 49 02 137 59 10.1002/lsm.22637 28220946 PMC5819602

[22] Sarmento A C Lírio J F Medeiros K S Marconi C Costa A P Crispim J C Physical methods for the treatment of genitourinary syndrome of menopause: a systematic review Int J Gynaecol Obstet 2021 153 02 200 19 10.1002/ijgo.13561 33354773

[23] Knight C Logan V Fenlon D A systematic review of laser therapy for vulvovaginal atrophy/genitourinary syndrome of menopause in breast cancer survivors Ecancermedicalscience 2019 13 988 10.3332/ecancer.2019.988 32010212 PMC6974376

[24] Rahn D D Carberry C Sanses T V Mamik M M Ward R M Meriwether K V Vaginal estrogen for genitourinary syndrome of menopause: a systematic review Obstet Gynecol 2014 124 06 1147 56 10.1097/AOG.0000000000000526 25415166 PMC4855283

[25] Cody J D Jacobs M L Richardson K Moehrer B Hextall A Oestrogen therapy for urinary incontinence in post-menopausal women Cochrane Database Syst Rev 2012 10 CD001405 10.1002/14651858.CD001405.pub3 23076892 PMC7086391

[26] Bachmann G Santen R J Genitourinary syndrome of menopause (vulvovaginal atrophy): treatment [Internet] 2021[cited 2021 Oct 30]. Available from:https://www.uptodate.com/contents/genitourinary-syndrome-of-menopause-vulvovaginal-atrophy-treatment

[27] Vesna A Neli B Benefit and safety of 28-day transdermal estrogen regimen during vaginal hysterectomy (a controlled trial) Maturitas 2006 53 03 282 98 10.1016/j.maturitas.2005.05.012 16011883

[28] ACOG Committee Opinion No. 659: the use of vaginal estrogen in women with a history of estrogen-dependent breast cancer Obstet Gynecol 2016 127 03 e93 6 10.1097/AOG.0000000000001351 26901334

[29] Faubion S S Larkin L C Stuenkel C A Bachmann G A Chism L A Kagan R Management of genitourinary syndrome of menopause in women with or at high risk for breast cancer: consensus recommendations from The North American Menopause Society and The International Society for the Study of Women's Sexual Health Menopause 2018 25 06 596 608 10.1097/GME.0000000000001121 29762200

[30] American College of Obstetricians and Gynecologists' Committee on Gynecologic Practice Committee Opinion No. 659: the use of vaginal estrogen in women with a history of estrogen-dependent breast cancer [Internet] 2016(Reaffirmed 2020) [cited 2021 Oct 30]. Available from:https://www.acog.org/clinical/clinical-guidance/committee-opinion/articles/2016/03/the-use-of-vaginal-estrogen-in-women-with-a-history-of-estrogen-dependent-breast-cancer

